# Highly-Integrated Hydraulic Smart Actuators and Smart Manifolds for High-Bandwidth Force Control

**DOI:** 10.3389/frobt.2018.00051

**Published:** 2018-06-14

**Authors:** Victor Barasuol, Octavio A. Villarreal-Magaña, Dhinesh Sangiah, Marco Frigerio, Mike Baker, Robert Morgan, Gustavo A. Medrano-Cerda, Darwin Gordon Caldwell, Claudio Semini

**Affiliations:** ^1^Department of Advanced Robotics, Istituto Italiano di Technologia, Genoa, Italy; ^2^Moog (United Kingdom), Tewkesbury, United Kingdom; ^3^Faculty of Engineering Sciences, KU Leuven, Leuven, Belgium

**Keywords:** hydraulic actuation, legged robot, servo valve, integrated smart actuator, dynamic modeling, control architectures, additive manufacturing, low leakage

## Abstract

Hydraulic actuation is the most widely used alternative to electric motors for legged robots and manipulators. It is often selected for its high power density, robustness and high-bandwidth control performance that allows the implementation of force/impedance control. Force control is crucial for robots that are in contact with the environment, since it enables the implementation of active impedance and whole body control that can lead to a better performance in known and unknown environments. This paper presents the hydraulic *Integrated Smart Actuator (ISA)* developed by Moog in collaboration with IIT, as well as smart manifolds for rotary hydraulic actuators. The ISA consists of an additive-manufactured body containing a hydraulic cylinder, servo valve, pressure/position/load/temperature sensing, overload protection and electronics for control and communication. The ISA v2 and ISA v5 have been specifically designed to fit into the legs of IIT’s hydraulic quadruped robots HyQ and HyQ-REAL, respectively. The key features of these components tackle 3 of today’s main challenges of hydraulic actuation for legged robots through: (1) built-in controllers running inside integrated electronics for high-performance control, (2) low-leakage servo valves for reduced energy losses, and (3) compactness thanks to metal additive manufacturing. The main contributions of this paper are the derivation of the representative dynamic models of these highly integrated hydraulic servo actuators, a control architecture that allows for high-bandwidth force control and their experimental validation with application-specific trajectories and tests. We believe that this is the first work that presents additive-manufactured, highly integrated hydraulic smart actuators for robotics.

## 1. Introduction

Hydraulic actuation is the most widely used alternative to electric motors for legged robots and manipulators. It is not only the high power-to-weight ratio and the high control bandwidth (Mavroidis et al., 1999; Siciliano and Khatib, 2007) that make hydraulic actuators interesting. Other major advantages are related to the inherent properties of hydraulic oil that acts as a lubricant as well as cooling liquid. Despite these advantages, hydraulic actuation suffers from a number of short-comings. The recently published *Survey on Control of Hydraulic Robotic Manipulators with Projection to Future Trends* (Mattila et al., 2017) mentions two of them: First, the difficult controller design due to the nonlinearity of the hydraulic system dynamics and second, the low energy efficiency of traditional closed-loop hydraulic systems. Additionally, compact hydraulic actuation components are rare on today’s market and their compact integration into articulated robots is challenging (Semini et al., 2011). Hydraulic servo actuators have been used for several decades in legged robots. Marc Raibert’s early hopping robots (e.g., the 3D one-legged hopping machine) were driven by hydraulic actuators that combined a low friction cylinder, position sensor, velocity sensor and pressure control servo valve (Raibert, 1986). Raibert continued using similar custom actuators for the robots developed by his company Boston Dynamics, Inc. (BDI). The legs of *BigDog*, for example, are powered by a custom hydraulic actuator with a Moog Series 30 servo valve^[1]^, cylinder, load cell and potentiometer (Buehler et al., 2005). The servo valves allowed the actuators to be controlled in force as well as in position. Subsequently developed BDI robots like LS3, Cheetah, Wildcat, ATLAS and Spot use hydraulics^[2]^, but no detailed information about the servo actuators is available. IIT’s HyQ robot uses a custom hydraulic servo actuator that consists of a Moog E024 servo valve^[3]^, a Hoerbiger cylinder, a custom hydraulic manifold, 2 pipes, a load cell, an absolute and relative joint encoder, and electronics for sensors and valve amplifiers (Semini et al., 2011). Boaventura et al. presented high-performance force control on these actuators (Boaventura et al., 2015). Another force-controlled hydraulic actuation unit was developed by Hyon et al. for a light-weight hydraulic leg (Hyon et al., 2013) that was later used as the basis for the actuation of the joints of a hydraulic humanoid robot called *TaeMu* (Hyon et al., 2017). The actuator units use PSC AS110 servo valves^[4]^. The *BabyElephant* robot (Gao et al., 2014) is powered by custom-made hydraulic actuators called *Hy-Mo*. These actuators consist of a hydraulic cylinder, an electric motor to move the spool of the valve and pressure sensors to measure the two chamber pressures (Wang et al., 2014). Another example is the hydraulic force control unit of the hydraulic quadruped robot BITDOG (Lu et al., 2015). The servo actuator consists of a hydraulic servo valve, hydraulic actuating cylinder, displacement sensors, pressure sensors and shock absorber. The actuator’s active compliance controller was presented in Lu et al. (Lu et al., 2015). The ROBOCLIMBER is a 4,000 kg quadruped machine with force-controlled prismatic legs. Montes et al. present various force control strategies for the hydraulic cylinders (Montes and Armada, 2016).

Besides academic prototypes, there are also a few hydraulic servo actuators available on the market, such as the Moog *A085 Series Servo Actuators*^[5]^ that combine high performance cylinders, linear feedback devices and servo valves in one assembly. These actuators were recently installed on a Menzi Muck walking excavator to implement active chassis balancing with force controlled cylinders (Hutter et al., 2017). The force was estimated with hydraulic pressure sensors inside the two cylinder chambers. Other commercial examples are the linear and rotary actuators developed by KNR systems (Kim et al., 2014) that feature KNR series KSV070 servo valves^[6]^. A combination of KNR actuators and Star Hydraulics series 200 servo valves^[7]^ are used for the hydraulic quadruped robot Jinpoong developed by the Korea Institute of Industrial Technology (KITECH) (Cho et al., 2016).

A related class of integrated hydraulic actuators are electro-hydrostatic actuators (EHA) that combine an electric motor, hydraulic pump, small tank, and rotary/linear actuator into one unit. Two examples from academia are the following: Alfayad et al. have recently presented the *IEHA - Integrated Electro Hydraulic Actuator* for a hydraulic humanoid called *Hydroid* (Alfayad et al., 2011). Kaminaga et al. have been developing EHAs for robotic hands and recently for the humanoid robot called *Hydra* (Kaminaga et al., 2014). The remainder of this paper will exclusively focus on actuators driven by servo valves.

Most high-performance, hydraulic legged robots (see list above for examples) rely on miniature servo valves to control the actuators in their legs. The fast response and high control bandwidth of servo valves, when compared to other types of valves, allow the implementation of force/torque control, which is an important building block to achieve robust locomotion (Semini et al., 2015). The high bandwidth of servo valves, however, has its price: The pilot stage has a continuous internal leakage (the so-called *tare leakage*) that routes part of the flow directly from the pressure port back to the return port. This *wasted* flow leads to a generally low energy efficiency of servo hydraulics. Note that the tare leakage does not include the spool null leakage.

Table 1 compares the most important specifications of the above-mentioned servo valves. The last column show the specifications of the ISA v5 that will be introduced next.

**Table 1:** Comparison of the specifications of some servo valves used in hydraulic legged robots.

**Property**	**Moog E024**	**Moog 30**	**KNR KSV070**	**Star-Hydraulics 200**	**Moog ISA v5 valve**
Maximum operating pressure (MPa)	21.0	27.5	21.0 (assumed)	31.5	20.7
Rated flow at 7MPa ΔP (l/min)	7.5	6.8	5.5	7.0	7.5
−3 dB bandwidth at 25% input (Hz)	>250	>200	>60	200*	>100
Tare leakage at 20.7 MPa (l/min)	<0.3	<0.34	0.33*	<0.8 total int. leak. at 140 bar	<0.06
Weight (g)	92	190	178	230	built-in

All values are taken from the data sheets mentioned in the footnotes 1 to 6. An asterisk (*) indicates that a value has been estimated from a plot.

**Figure 1:**
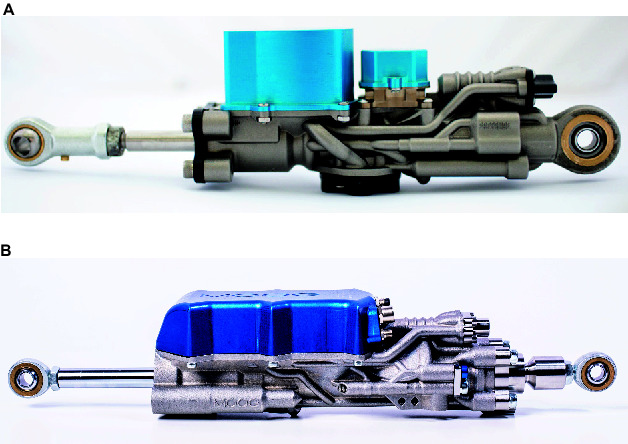
Pictures of two Integrated Smart Actuators (ISA) developed by Moog in collaboration with IIT. **(A)** ISA v2: custom-sized for HyQ hip/knee joints; **(B)** ISA v5: custom-sized for HyQ-REAL knee joints.

This paper presents two versions of the new Integrated Smart Actuators (ISA) developed by Moog in collaboration with IIT, as well as a smart manifold on a rotary actuator. The ISA consists of a hydraulic cylinder, servo valve, various sensors, overload protection and electronics for control and communication. Its body is additively manufactured (AM) in a titanium alloy, allowing a very compact design with integrated flow paths and wire channels. The two presented versions ISA v2 and ISA v5 (Figure 1A,B) have been specifically designed to fit into the legs of IIT’s hydraulic quadruped robots HyQ (Semini et al., 2011) and the newest version HyQ-REAL (under construction). The last column of Table 1 shows the key specifications of the new ultra-low leakage valve inside the ISA v5 and smart manifolds (see Section 2).

The main contributions of this paper are the derivation of the representative dynamic models of these highly integrated hydraulic servo actuators, a control architecture that allows for high-bandwidth force control and their experimental validation with application-specific trajectories and tests. The key features of these components tackle the disadvantages of hydraulic actuation for legged robots through: (1) built-in controllers running inside integrated electronics, (2) low-leakage servo valves, and (3) compactness thanks to metal additive manufacturing. We believe that this is the first work that presents additive-manufactured, highly integrated hydraulic smart actuators for robotics. The ISA has been mentioned in a paper on additive manufacturing by Moog’s Guerrier et al. presented at the 2016 conference on Recent Advances in Aerospace Actuation Systems and Components (Guerrier et al., 2016). A short overview of the ISA has been presented during the IROS 2016 workshop on *The Mechatronics behind Force/Torque Controlled Robot Actuation: Secrets and Challenges* (Semini et al., 2016).

This paper is structured as follows: Section 2 presents the main features and specifications of the ISA and smart manifolds. Section 3 describes the control architecture running on the actuator’s embedded ARM processor, and Section 4 explains the mathematical model of the actuator dynamics. Section 5 presents the results of simulation and experiments, followed by the conclusions in Section 6.

## 2. ISA and Smart Manifold Features

**Figure 2:**
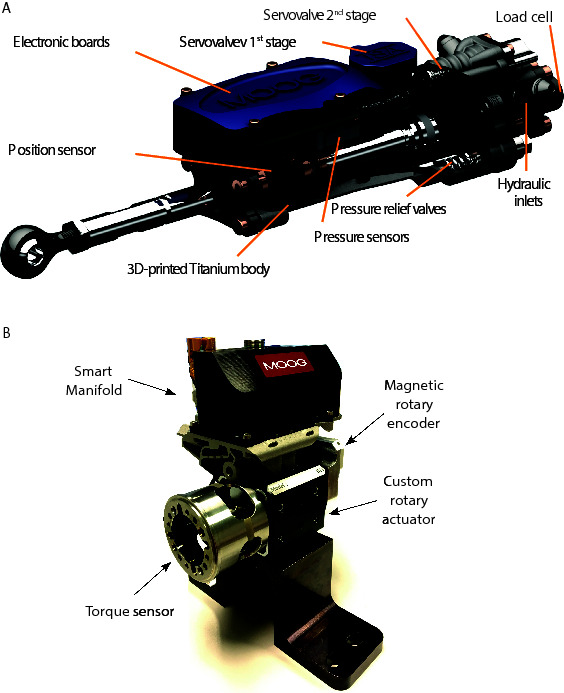
Integrated smart actuators: **(A)** CAD rendering of the ISA v2 with cut-out section to illustrate the main features of the actuator and integrated components (illustration also representative of the ISA v5). **(B)** Smart manifold mounted on a custom made rotary actuator. A custom torque sensor and the absolute position sensor are interfaced with the smart manifold.

The linear actuator ISA v2 was developed with the goal of integrating standard hydraulic components by means of additive manufacturing to create an optimized actuator unit. The ISA v2 has a high response valve and the most important sensors for position/force control and self-protection (temperature and mechanical impacts). Figure 2A shows a CAD rendering of the ISA v2 with a section to illustrate the main features of the actuator and integrated components.

**Figure 3:**
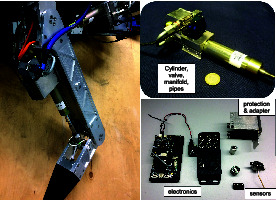
Picture of a leg of HyQ (left) with two separate pictures showing the components that constitute a linear actuator unit of the robot (right).

The use of ISAs in a legged robot has several advantages on system level: First, it reduces the overall complexity of the machine, since the various actuator components are combined into one device. Sensor wires are routed inside the AM body and several components are merged into the same electronic board (e.g., microcontroller, valve amplifier, Inertial Measurement Unit (IMU), temperature sensor). Fewer and shorter wires result in higher reliability and less signal noise. Second, it reduces the total robot weight, and increases its ruggedness. For an illustrative comparison, Figure 3 shows the linear actuator units of a HyQ leg with all the components that belong to one unit. Note that the shown A/D converter and communication electronic boards - shown on the left of the bottom-right picture - are connecting to 3 actuators in total. The black box to its right hosts the electronics for 6 valve amplifiers. The total estimated weight of the components of 1 actuator unit (excluding the electronics) is 1.3 kg. The corresponding ISA v2 on the other hand weighs only 0.92 kg and additionally includes pressure relief valves for overload protection, as well as all electronics to close local control loops.

The need for more efficient actuators in autonomous robots triggered the redesign of the linear actuator ISA v2 to achieve the version ISA v5, with a compromise between high performance and energy wasting.

The combination of additive manufacturing and standard parts allows for customisation of actuators and retain all of the functionalities that of the ISA. This idea has been used to create the smart manifolds to provide most of the ISA technology to custom made rotary actuators as shown in Figure 2B.

In this section we present the most important features and specifications, regarding the mechanical and electronic components, of the integrated smart actuators and smart manifolds.

### 2.1. Integrated Servo Valve for High Performance (ISA V2)

**Figure 4:**
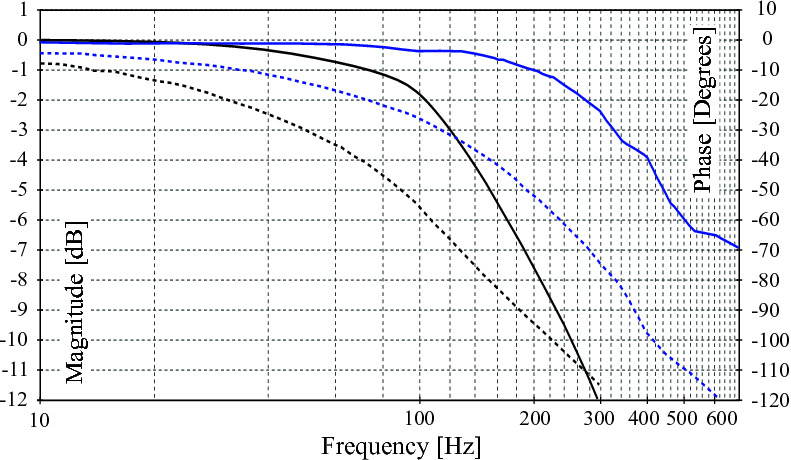
Frequency response for the high response valves integrated in the ISA v2 and ISA v5 (solid lines for magnitude and dashed lines for phase responses). The lines show the frequency response for the high response valve (HRV) of the ISA v2 presenting cut-off frequency around 320 Hz. The black lines show the frequency response for the ultra-low leakage valve (ULLV) integrated in the ISA v5 presenting a cut-off frequency around 120 Hz.

The integrated servo valve is a derivative of the high performance version of the Moog E024 series sub-miniature servo-valves (MOOG Inc, 2015) (valve used in the HyQ robot). The high bandwidth of around 320 Hz allows high performance force and position control, as previously demonstrated in (Boaventura et al., 2015). The frequency response for the high response valve (HRV) used in the ISA v2 is shown in Figure 4.

### 2.2. Integrated Servo Valve for High Performance with Improved Efficiency (ISA V5)

**Figure 5:**
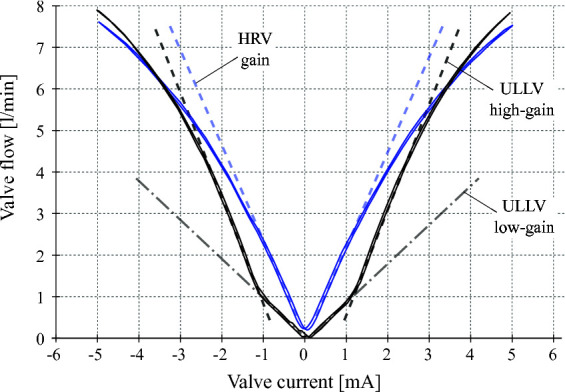
Flow gain curves for the HRV (blue line) and ULLV (black line). The HRV presents a constant flow increasing of about 2.3 l/min/mA until near 2 mA, when the valve gain start decreasing. The ULLV, instead, presents a low flow gain of 0.4 l/min/mA and a high flow gain of ±2.5 l/min/mA inside and outside the current range of ±1.2 mA, respectively. Note: these curves were obtained from experimental measurements considering the valve redundant coils (2 coils) in series. The ISA and smart manifolds integrate these valves with the coils connected in parallel to increase the operation safety. In this case, the scale of the current is doubled and the current command ranges from ±10 mA.

**Figure 6:**
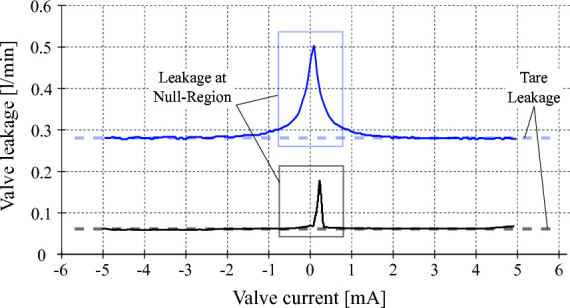
Valve tare and null-region leakages for the high response valve (blue curve) and for the ultra-low leakage valve (black curve). Experimental measurements show about 0.06 l/min and 0.28 l/min of tare leakage for the ULLV and HRV valves, respectively. Leakage peaks at spool null region are about 0.5 l/min for the HRV and 0.17 l/min for the ULLV. Considering an operating pressure supply of 20.7 MPa, the ULLV may save about to 41 W due to tare leakage and up to 112 W at the spool null region.

ISA v5 incorporates an ultra-low leakage valve (ULLV) technology to considerably reduce throttling losses and improve the efficiency of the overall unit. The pilot stage of the valve is modified to improve tare leakage and still have high bandwidth of greater than 100 Hz as shown in Figure 4. The second stage peak leakage is reduced with valve overlap and dual gain flow slots. Figure 5 compares the flow gain of the valve configuration to that of the high response valve used in ISA v2. Here the v5 has low-flow gain near null and high-gain stroke greater than 25%. The low gain flow slots near null considerably reduces losses and reduces nonlinearity in the flow curve as opposed to purely overlapped valves. Figure 6 compares the leakage flow of ISA v5 (ULLV) to that of ISA v2 (HRV). When the actuator is stationary the leakage of ISA v5 is approximately 36% of ISA v2. This results in a power saving of approximately 112W per actuator.

### 2.3. Unequal Area Flow Slots (ISA V5)

ISA v5 has unequal area flow slots which match the area ratio of the actuator. This results in constant actuator velocity during retraction and extension and removes the need for gain scheduling based on the piston motion direction.

### 2.4. Integrated Sensors for Position, Pressure and Force Measurement

A 1Mbps absolute position sensor is used to measure the position of the piston rod. The two cylinder chamber pressures of the actuator are measured using strain gauge based miniature pressure sensors. Actuator force is measured using a strain gauge based load cell integrated in the actuator tail stock. The electronic board additionally includes an IMU and ports for optional sensors.

### 2.5. Integrated Electronics to Control Actuator Position/Force

The design of the Remote Electronics Unit (REU) follows Moog’s commercial aircraft flight control standards. It closes several control loops (see Section 3) on an ARM processor and offers various communication options such as, e.g., EtherCAT, CAN and Serial bus.

### 2.6.Valve Spool Feedback

The smart manifold has a mechanical feedback valve (MFB valves), i.e., the spool position is indirectly controlled from the valve current command. The ISA v2 and v5, instead, have electric feedback valves (EFB valve) and the spool position can be fed back in a specific control loop. Electric feedback technology allows for a valve response less sensitive to the null bias (which is dependent on the return and supply pressures and on the oil temperature).

### 2.7. Integrated Overload Protection

Pressure relief valves limit the pressure inside the two cylinder chambers. These valves vent to return if the chamber pressures reach 22 MPa thus resulting in an effective and repeatable overload protection.

Tables 2, 3 show the most relevant mechanical and electrical features of the ISAs and smart manifolds, respectively.

**Table 2:** Mechanical specifications of the ISA v2, v5 and smart manifold for rotary actuator.

**Actuator Property**	**ISA v2**	**ISA v5**	**Smart manifold(custom made)**
Cylinder bore diameter (linear)	16 mm	21.5 mm	-
Rod diameter (linear)	12 mm	12 mm	-
Length (retracted – linear)	235 mm	299 mm	-
Vol. displacement (rotary)	-	-	0.15 cc/deg
Total stroke	80 mm	100 mm	100 deg
Dry weight	920 g	1,600 g	2,100 g
Stall load	4,000 N	7,500 N	170 Nm
Operating pressure	20.7 MPa	20.7 MPa	20.7 MPa
Operating temperature	[−30 + 80] °C	[−30 + 80] °C	[−30 + 80] °C
Valve spool feedback	Electric	Electric	Mechanic

**Table 3:** Electrical specifications of the boards for the ISA (both v2 and v5) and the smart manifold.

**Property**	**ISA**	**Smart manifold**
Operating voltage	24 V	24 V
Max current	125 mA	125 mA
Sensor sampling frequency	10 kHz	10 kHz
ADC resolution	12 bit	12 bit
Encoder resolution	1 μm	$6.9\times 10^{-4}$ deg
Encoder Baud rate	1 Mbps	1 Mbps

## 3. Control Architecture

This section describes the standard control loops available on the Remote Electronics Unit (REU) that is integrated in the smart actuators (i.e., ISA v2, ISA v5 and smart manifolds). The REU, also called thumb-REU for its small board size, has standard nested control loops that can be configured according to the actuator hardware. For example, for the more innovative lines (the ISA v2 and v5 with electric feedback valves), it is available a control block with a set of functionalities to control the spool position. For the case of smart manifolds, which have mechanic feedback valves and no spool position measurements, such control block can be reconfigured to control another state of the actuator. By available control block we mean a block with a set of control functionalities that can be used by the control designer. Such functionalities are, e.g.,: PID controllers, feedforward terms, feedback terms, filters, selection of control loop frequency, saturation functions, anti wind-up for integral actions, gain scheduling, offsets, and logic blocks for nonlinear compensations.

The standard firmware of the thumb-REU provides four control blocks where each one can be configured to be associated to one of the following actuator variables: piston/rotor position, pressure difference between the actuator chambers (i.e., ΔP), actuator force/torque, spool position and valve current. Each control block, though, can be activated or deactivated at will, to achieve different control strategies. For example, to perform pure actuator position control, force/torque control, or even activate only the valve current loop to use the thumb-REU as a simple valve amplifier.

**Figure 7:**
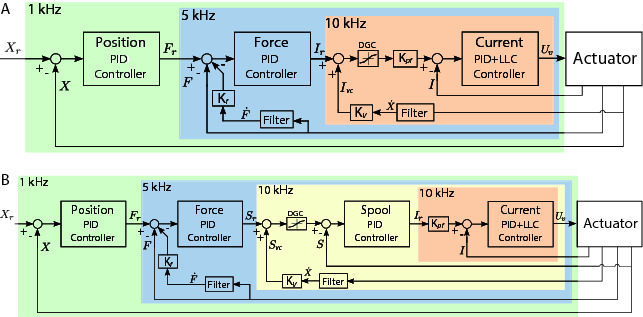
Control diagram illustrating the arrangement of control blocks for each actuator. **(A)** Control architecture for the smart manifold mounted on the custom rotary actuator with three nested loops: rotor position control, torque control (at load level using a torque sensor), and the valve current control. **(B)** Control architecture for the ISA v5 with four nested loops: piston position, force control (at load level using a load cell), spool position control, and valve current control. The control loop frequency selected for each block is indicated on the top-left corner of each respective translucent color box. A velocity compensation term ($K_{v}\dot{X}$) and a valve current compensation block DGC (due to the valve dual-gain feature) is used in both control architectures. Details on the PID actions and additional terms are described in Section 5.

Figure 7 shows a simplified view of each control architecture (arrengement of the available control blocks) that was used to test the ISA v5 (shown in Figure 1B) and the smart manifold with the custom made rotary actuator (shown in Figure 2B). The frequency of the control loops range from ~1 kHz for the piston/rotor position to ~10 kHz for the valve current. Both control diagrams show a force loop with an outer position loop that leads to an impedance control, which is an essential strategy for legged systems and will be used in the HyQ-REAL robot.

Both control architectures shown in Figure 7 have a force/torque control loop with feedback of the force/torque measured at the load level (instead of performing hydraulic force/torque control), what minimizes tracking issues due to the internal hydraulic actuator friction. To compensate for the pressure dropping due to the piston/rotor motion, a velocity compensation term is used to inject extra flow into the chambers. The extra current command from the velocity compensation is modulated according to the dual-gain feature of the ULLV to avoid a flow over compensation, at the valve region of high gain, that can make the overall control loop to be unstable.

For the case where the velocity compensation is introduced at the level of the spool control loop (see Figure 7B), $K_{v}$ is tuned to take into consideration the relationship between the spool opening and the valve flow. Details on the PID actions, of each block, and the additional terms considered for the test of the actuators are described in Sec. 5.

### 3.1. Current Loop Tuning

The current control loop of the smart actuators is the innermost loop and it is the loop that has negligible coupling with the load dynamics. Moreover, such decoupling allows for a control tuning only dependent on the internal dynamics of the valve, i.e., the valve coil dynamics.

**Figure 8:**
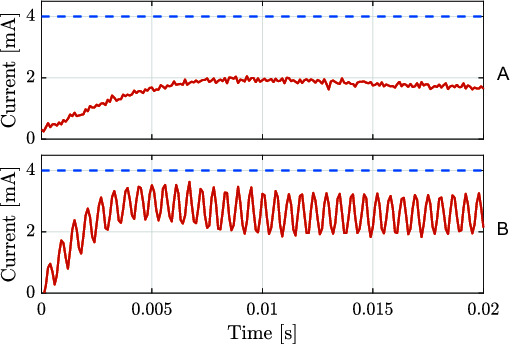
Valve current loop responses (solid red lines) for a current step input (dashed blue lines) of 4 mA: **(A)** open-loop current response and **(B)** closed-loop current response considering a proportional controller with $K_{p}$ = 4.55 V/mA. The static gain $K_{sg}$ extracted from **(A)** has approximated value of 0.4.

Given the importance of the current loop dynamics for the outer loops, we design a current controller that aims for a small phase lag between the desired and actual valve current. We propose a control structure in a 2-DOF (degrees of freedom) configuration where: (1) the closed-loop has the forward path composed of a proportional action, in series with a lead-lag compensation of second-order; and (2) a gain $K_{pf}$ applied on the demand to reduce steady-state errors. The gain $K_{pf}$ applied on the demand compensates for the steady-state error due to the absence of integral action (avoided to reduce the phase lag in the response). The lead-lag compensation is implemented to supress high-frequency resonance modes of the valve coil that are excited as the proportional action is increased. Figure 8A shows a step input response test performed in open-loop to obtain the static gain, here denoted as $K_{sg}$ between the desired current and the actual current. Figure 8B, instead, shows the apearance of a high-frequency resonance mode, around 1.8 kHz, for a step input response test when the current loop is closed with a certain proportinal action.

The transfer function of the current controller, named as PID + LLC in Figure 7, is described in the Laplace domain as:

(1) $$C(s)=K_{p}\frac{w_{lag}^{2}(s^{2}+2\zeta_{lead}w_{lead}s+w_{lead}^{2})}{w_{lead}^{2}(s^{2}+2\zeta_{lag}w_{lag}s+w_{lag}^{2})}$$

where $K_{p}$ is the proportional gain of the current loop, $w_{lead}$ and $w_{lag}$ are the natural frequencies for the lead and lag compensations, respectively, and $\zeta_{lead}$ and $\zeta_{lag}$ are the damping ratios for the lead and lag compensations, respectively.

 The gain $K_{pf}$ can be computed from $K_{p}$ and $K_{sg}$ as:

(2) $$K_{pf}=\frac{1+K_{p}K_{sg}}{K_{p}K_{sg}}$$

The values used for the current loops of both the ISA and the Smart Manifold are described in (Table 4). With such tuning one obtains a current control loop with response time of less than 2 ms.

**Table 4:** Controller gains implemented in the system.

Loop	Gain	Smart manifold (rotaty)	ISA V5 (linear)
Current	$K_{p}$	16 V/mA	16 V/mA
	$K_{i}$	-	-
	$K_{d}$	-	-
	$K_{pf}$	1.15	1.15
	$\omega_{lead}$	10,681 rad/s	10,681 rad/s
	$\omega_{lag}$	2,513.3 rad/s	25,133 rad/s
	$\zeta_{lag}$	0.1	0.1
	$\zeta_{lead}$	0.65	0.65
Spool	$K_{p}$	-	0.4 mA
	$K_{i}$	-	-
	$K_{d}$	-	-
Force/torque	$K_{p}$	0.04 mA/Nm	0.02 mm/N
	$K_{i}$	7 mA/Nms	0.85 mm/Ns
	$K_{d}$	-	-
Position	$K_{p}^{*}$	300 Nm/rad	20,000 N/mm
	$K_{i}$	-	-
	$K_{d}^{*}$	20 Nms/rad	9,500 Ns/mm
Velocity compensation	*K*_*v*_	0.65 mA/s	240 s
Force damping gain	*K*_*f*_	0.004 s	0.006 s

Gains marked with an asterisk (*) may change depending on the task.

## 4. Actuator Modeling

In this section the dynamics of the hydraulic actuators are described^[8]^. The main goal is to provide a representative mathematical model, from the valve to the load dynamics, in order to: (1) complement the simulation of the rigid body dynamics of a legged system making it more realistic; (2) help designing new control strategies to improve force/torque tracking performance and robustness; and (3) to serve as a tool to understand the impact of the mechanical design of parts on the controller performance (e.g., friction and backlash). In Section 5 the model is used to simulate the experiments where the actuator is tested under representative conditions of legged systems.

The power conversion in hydraulic actuation relies on the transmission of fluid by means of a pump to a hydraulic actuator. The role of the actuator is to transform back the hydraulic energy into mechanical energy, which is then transmitted to a mechanical device (e.g., a robot leg).

In the following paragraphs a description of the pressure, the flow, the valve spool and the load dynamics of hydraulic actuators is given. The rate of change of the pressure with respect to time in a given chamber is represented by the so-called *Continuity Equation*, which can be used to obtain the pressure dynamics for a linear hydraulic actuator as

(3) $${\dot{P}}_{A}=\frac{\beta_{eff}}{V_{0A}+A_{A}x_{p}}\left(Q_{A}-A_{A}{\dot{x}}_{p}-C_{li}\left(P_{A}-P_{B}\right)\right),$$

(4) $${\dot{P}}_{B}=\frac{\beta_{eff}}{V_{0B}+A_{B}\left(L_{p}-x_{p}\right)}\left(Q_{B}+A_{B}{\dot{x}}_{p}-C_{li}\left(P_{B}-P_{A}\right)\right),$$

where $P_{A}$ and $P_{B}$ are the pressures inside the chambers, $V_{0A}$ and $V_{0B}$ are the dead volumes coming from the valve inside the chambers, $A_{A}$ and $A_{B}$ the piston/vane areas where pressure is exerted in each of the chambers, $Q_{A}$ and $Q_{B}$ are the flows inside the chambers, $x_{p}$ is the piston position, $L_{p}$ represents the cylinder stroke length and $C_{li}$ is a coefficient related to leakage. The *Bulk Modulus* is a measure for the compressibility of the fluid (commonly denoted by β). Due to flexibility of the compartments that contain the fluid and undissolved air pollution in the fluid, changes in the volume may not be captured by β. In this case, the Bulk Modulus β is replaced by the *Effective Bulk Modulus*$\beta_{eff}$.

From Equations (3) and (4), the pressures in each of the chambers can be obtained and the hydraulic force delivered by the piston is given by

(5) $$f_{h}=A_{A}P_{A}-A_{B}P_{B}.$$

The pressure dynamics equations for a rotary actuator can be obtained in a similar fashion with respect to the linear case by replacing both piston areas $A_{A}$ and $A_{B}$ with the volumetric displacement of the motor $D_{m}$, the piston position $x_{p}$ with the angular position of the rotor $\theta_{a}$ and the cylinder stroke length $L_{p}$ with the range of the motor $L_{m}$. The hydraulic torque delivered by the rotor can also be obtained from the pressure dynamics as

(6) $$\tau_{H}=D_{m}(P_{A}-P_{B}).$$

**Figure 9:**
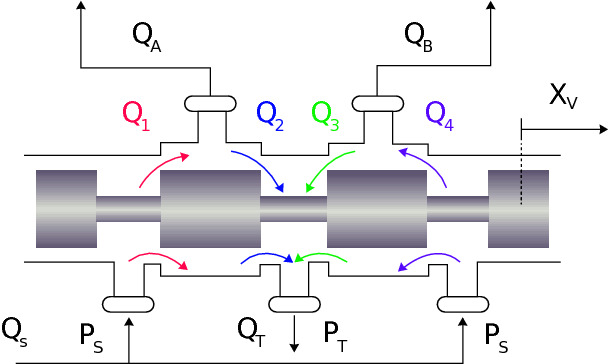
Schematic drawing of a valve that controls the flows $Q_{A}$ and $Q_{B}$ in the actuator chambers. $P_{T}$ and $P_{S}$ represent the return and supply pressure, respectively, $Q_{T}$ and $Q_{S}$ are the return and supply flows, respectively, and $Q_{i}$ for i=1,2,3,4 are valve port flows.

It can be noticed that the pressure dynamics not only depend on the position of the piston or the rotor, but they also depend on the flows going into each of the chambers of the actuators. These flows ($Q_{A}$ and $Q_{B}$) can be obtained from the dynamics of the valve. However, the valve is one of the elements that renders the modeling and control of hydraulic systems more complex, due to its nonlinear behavior. Figure 9 shows a schematic drawing of a valve which controls the flows $Q_{A}$ and $Q_{B}$. The spool position determines the amount of flow that goes through the orifices of the valve.

In the case of our experimental setups, a novel dual-gain Ultra Low Leakage Valve (ULLV) developed by Moog and IIT is being used. The spool position is controlled by means of an input current. The relation between input current and spool position is usually modeled as a second order system [see (Boaventura et al., 2015)] of the form

(7) $$X_{v}\left(s\right)=\frac{\omega_{n}^{2}K_{spool}}{s^{2}+2\zeta \omega_{n}s+\omega_{n}^{2}}I\left(s\right),$$

where $X_{v}(s)$ represents the spool position in the Laplace domain, I(s) is the valve current in the Laplace domain, $K_{spool}$ relates the current input with the spool position in steady state, $\omega_{n}$ is the natural frequency of the spool and ζ is the damping coefficient of the spool. However, for the ULLV a frequency domain analysis (depicted in Figure 4) showed higher order, which have been fit to a third-order transfer function given by

(8) $$X_{v}\left(s\right)=\frac{\omega_{n}^{2}K_{loop}}{\left(s^{2}+2\zeta \omega_{n}s+\omega_{n}^{2}\right)s+\omega_{n}^{2}K_{loop}}X_{v}^{r}\left(s\right)$$

where $x_{v}^{r}$ is the spool position reference, $K_{loop}$ = 540 rad/s, $\omega_{n}$ = 6132 rad/s and ζ=1.5. The relationship between the spool position reference $X_{v}^{r}(s)$ and the spool current command $I_{r}\left(s\right)$, in Laplace domain, are approximated by the following equations:

(9) $$X_{v}^{r}\left(s\right)=K_{spool}I\left(s\right)$$

(10) $$I(s)=\frac{p_{c}}{s+p_{c}}I_{r}(s),$$

where $K_{spool}$ = 0.0356 mm/mA and $p_{c}$ = 2,000 rad/s is the pole of the first order systems that approximates the current loop response of the current controller described in Sec. 3.

With the definition of the spool dynamics, one can obtain the flow going through an orifice using the following expression:

(11) $$Q=k_{v}\left(x_{v}\right)x_{v}\sqrt{\Delta P},$$

where $k_{v}(x_{v})$ is the so-called *valve gain*, and it is a factor that depends on the discharge coefficient, orifice area gradient and the density of the fluid (Merritt, 1967). Normally, $k_{v}$ is computed based on experiments with nominal input current and nominal flow, making use of a flow-meter. In the case of the ULLV, plots relating the flow going through the valve with respect to the input current were provided. These plots are depicted in Figure 5. The plots indeed show clearly the zones were the gain value changes, in the case of the ULLV. The low and high values of $k_{v}$ were estimated from these plots.

The flows $Q_{A}$ and $Q_{B}$ obtained from the differences between $Q_{1}$, $Q_{2}$, $Q_{3}$ and $Q_{4}$ in Figure 9 are computed according to the following equations (considering a critically centered valve)

(12) $$Q_{A}=Q_{1}-Q_{2},$$

(13) $$Q_{1}=k_{v1}(x_{v})sg(x_{v})sign(P_{S}-P_{A})\sqrt{\left|P_{S}-P_{A}\right|},$$

(14) $$Q_{2}=k_{v2}(x_{v})sg(-x_{v})sign(P_{A}-P_{T})\sqrt{\left|P_{A}-P_{T}\right|},$$

(15) $$Q_{B}=Q_{4}-Q_{3},$$

(16) $$Q_{3}=k_{v3}\left(x_{v}\right)sg\left(x_{v}\right)sign\left(P_{B}-P_{T}\right)\sqrt{|P_{B}-P_{T}|},$$

(17) $$Q_{4}=k_{v4}(x_{v})sg(-x_{v})sign(P_{S}-P_{B})\sqrt{\left|P_{S}-P_{B}\right|},$$

where the function sg(x) is defined as:

(18) $$sg\left(x\right)=\left\{ \begin{array}{ll} x & \text{ for }x\geq 0\\ 0 & \text{ for }x<0, \end{array}\right.$$

and $P_{T}$ and $P_{S}$ are the return and supply pressures, respectively. The variable valve gains $k_{vi}(x_{v})$, with i=1..4, are equally modeled according to the ULLV flow gain curve shown in Figure 5 and Eq. 9 as:

(19) $$k_{vi}(x_{v})=\left\{ \begin{array}{ll} 6.667\times 10^{-6}\;m^{3}/s & \text{for }|x_{v}|<x_{dg}\\ 4.167\times 10^{-5}\;m^{3}/s & \text{for }|x_{v}|\geq x_{dg}, \end{array}\right.$$

where $x_{dg}$ is equal to 42.7 × 10^−3^ mm.

It is possible to consider fluid compressibility, elasticity of the fluid container and fluid resistance in the model, but these effects are highly nonlinear and are out of the scope of this paper.

Hydraulic force (in the case of linear actuators) or hydraulic torque (in the case of rotary actuators) is applied onto a mechanical device. In the case of this study, an experimental setup was built in order to test a custom-made rotary actuator integrated with a smart manifold. Additionally, the ISA v5 linear servo actuator was mounted on the Knee Flexion/Extension (KFE) joint of the hydraulically actuated leg of HyQ-REAL. These experiments are explained in Section 5.

## 5. Simulation and Experimental Results

**Figure 10:**
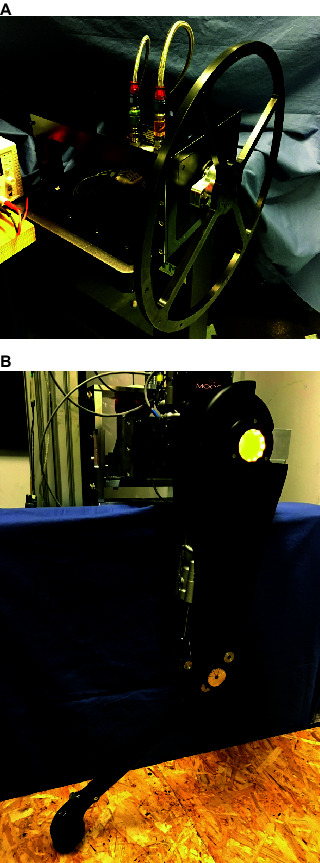
Pictures of the experimental setups of the smart manifold and the ISA v5: **(A)** Smart manifold mounted on custom made rotary actuator driving a wheel; **(B)** ISA v5 driving the knee joint of a HyQ-REAL leg.

To evaluate the performance of the ISA v5 and the smart manifold, two experimental setups were devised. The first setup consists of the smart manifold mounted on a custom made rotary actuator, which drives a metal wheel. This setup is shown in Figure 10A. For the second experimental setup, the ISA v5 was mounted on a leg of the HyQ-REAL to drive the KFE joint. This setup is depicted in Figure 10B. This section includes the simulation and experimental results on the rotary actuator setup (Figure 10A) and the experimental results on the KFE joint driven by the ISA v5 (Figure 10B).

The experiments were performed using the control strategy depicted in Figure 7 and Table 4 shows the specific values of the controller gains for each of the loops (unless it is indicated differently). It is worth mentioning that in the rotary actuator there is no spool loop. This is due to the fact that the connection of the first and second stage of the valve is done mechanically. On the other hand, the stages of the valve in the leg experimental setup is done through electric feedback, which gives rise to the spool control loop.

### 5.1. Integrated Smart Manifold and Rotary Actuator

The core of the test rig is made of three main components: the smart manifold, a custom-made rotary actuator and a set of sensors useful for control and analysis (position, torque, pressure, among others). All of these elements are shown in Figure 2B. This configuration of integrated electronics along with a rotary hydraulic actuator is suitable for actuating a legged system, for example, a Hip Abduction/Adduction (HAA) joint of a quadruped robot, such as the ones on HyQ-REAL.

**Figure 11:**
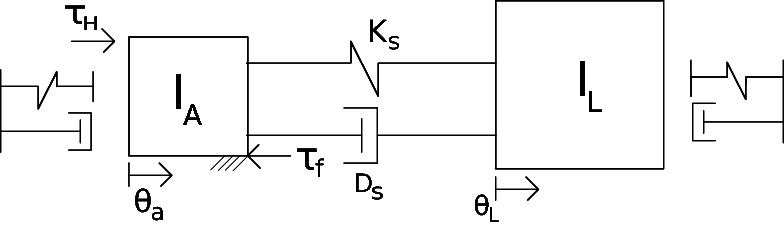
Schematic drawing of the linear equivalent of the rotational actuator experimental setup.

For the experiments, a metal wheel was mechanically connected to the rotary actuator, as it can be seen in Figure 10A. The weight, shape and dimensions of this wheel were designed in order to approximate the rotary inertia of the leg of HyQ-REAL, which is known from CAD data. The mathematical model of the test setup (along with the hydraulics model of Section 4) was obtained for simulation and control purposes. A schematic drawing of the linear equivalent of the model is shown in Figure 11 and its mathematical description is given by

(20) $$I_{a}{\overset{..}{\theta }}_{a}=\tau_{H}-\tau_{f}-K_{s}\left(\theta_{a}-\theta_{l}\right)-D_{s}\left({\dot{\theta }}_{a}-{\dot{\theta }}_{l}\right),$$

(21) $$I_{l}{\overset{..}{\theta }}_{l}=K_{s}\left(\theta_{a}-\theta_{l}\right)+D_{s}\left({\dot{\theta }}_{a}-{\dot{\theta }}_{l}\right)$$

where $I_{a}$ and $I_{l}$ represent the rotational inertia of the actuator rotor and the load, respectively, $\theta_{a}$ and $\theta_{l}$ correspond to the angular position of the actuator and the load, respectively, $\tau_{H}$ is the hydraulic torque coming from the actuator, $\tau_{f}$ is the friction torque acting on the mechanical rotor, $K_{s}$ stands for the torque sensor stiffness and $D_{s}$ is the damping present in the torque sensor.

In this model, the inertia of the torque sensor is being neglected since it is considered to be very small (approximately $2\times 10^{3}$ times smaller with respect to the load inertia). We consider as well that the sensor and the rotor are rigidly connected to each other (sensor position is equal to actuator position). We use a Lund-Grenoble Friction Model for the friction torque acting on the actuator rotor (Aberger and Otter, 2002), with a stiction level of $T_{s}=2$ and a Coulumb friction level of $T_{c}=1.9$, identified from experiments. Nonlinear effects such as backlash between the load and the sensor (actuator) position are considered using a similar approach as in (Merzouki et al., 2003). We also model the hard-stops of the system as spring-damper systems with no pulling effects. The proposed model is considered accurate enough for simulation and controller design. The specific values of the parameters of the model, such as stiffness and inertias, were obtained from CAD models and experiments. The experiments were designed to identify damping coefficients of the involved dynamic elements.

In addition, the inherent characteristics of the sensors (e.g., resolution and sampling frequency) and the frequencies implemented in the various loops are also included in the simulation. This information is taken from the datasheets provided by the manufacturers of the sensors and electronic boards. In further studies, variations on the system due to quantization errors or sampling frequencies can be analyzed using the proposed simulation. The motor parameters used in simulation are the ones of the custom made actuator provided by the manufacturer and are shown in Table 5. To obtain $V_{0A}$ and $V_{0B}$ the following expressions using the pipe volume $V_{p}$ are used:

**Table 5:** Rotary actuator parameters.

Property	Value
$D_{m}$	8.59 × 10^−6^ m^3^/rad
$L_{m}$	1.745 rad
$C_{li}$	0.22 lpm @ 200 bar
$V_{p}$	1 ml
$\beta_{eff}$	7 × 10^8^ N/m^2^

(22) $$V_{0A}=V_{p}+D_{m}\theta_{a},$$

(23) $$V_{0B}=V_{p}+D_{m}(L_{m}-\theta_{a}).$$

**Figure 12:**
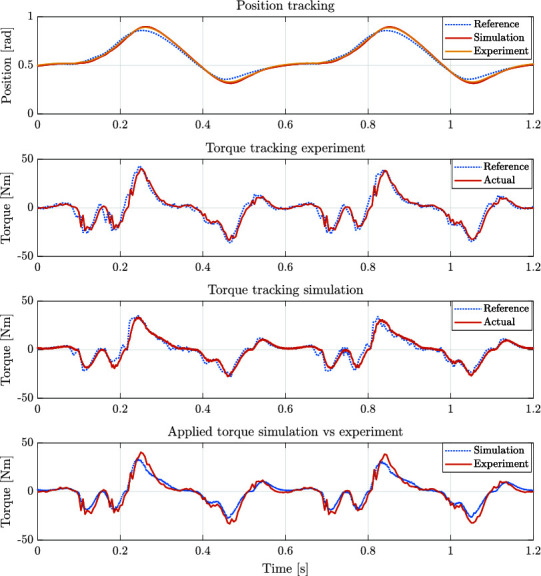
Position and torque tracking in both simulation and experiments for a reference trajectory of a rotary joint of the quadruped robot HyQ-REAL performing a trotting gait at a 1 m/s forward velocity.

For the first experiment, a reference signal for a rotary joint of a leg was obtained from simulation. The simulation corresponds to the robot HyQ-REAL performing a trotting gait with a forward velocity of 1 m/s. Figure 12 shows the position and torque tracking performance achieved, both during simulations and experiments. It is worth noting the resemblance between the generated signals both in simulation and experiments. We consider that the achieved performance is sufficient to show the capabilities of the designed actuators in robot locomotion applications. Performance can be further improved by including the nonlinearities related to the hydraulic force and the valve dynamics in the controller design in a similar fashion as in (Boaventura et al., 2015).

**Figure 13:**
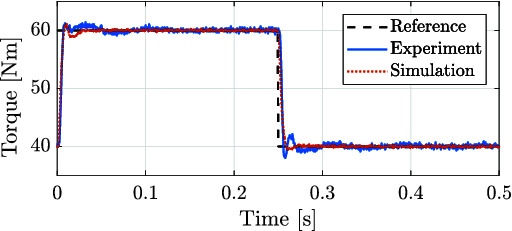
Torque applied for both the experimental (solid blue line) and simulated (red dashed line) corresponding to the tracking of a step signal with blocked joint load. The time response for 10% of the maximum output torque suggests a torque control bandwidth between 50 Hz and 100 Hz during blocked condition.

For the second experiment, the rotor actuator position was blocked, and a step torque reference was given in order to evaluate the torque tracking performance of the system. Figure 13 shows an example of the torque tracking during simulation and the experiments. The actuator eliminates the steady state error with a rise time of approximately 8 ms. These results match between simulation and experimental data. This step response suggests that the achievable bandwidth goes from 50 Hz to 100 Hz during blocked condition.

**Figure 14:**
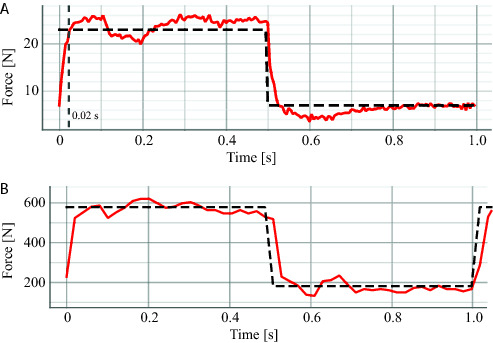
Force tracking for the ISA against the pallet. **(A)** small forces and **(B)** medium forces.

A difference between simulation and experiments can be noted in Figure 13, where low-amplitude oscillations are present before reaching steady state during experiments. One cause of this oscillatory behavior can be attributed to the contact model between the setup and the mechanical end-stop when the actuator is blocked. This assumption is supported by the fact that in the case of the rotary actuator, a stiff metal to metal contact was used to keep the motor in a constant position. This contact shows low-amplitude oscillations. In the case of the linear actuator experiments (shown in Figure 14) the contact was kept between metal and rubber. The compliance displayed by the rubber and its possible nonlinear stiffness, potentially increase the amplitude of the oscillation. This effect was also tested in simulation and the result coincide with this assumption. Added to the contact dynamics, a hunting effect originated due to the overlapping of the valve around the null position may also accentuate this oscillatory behavior.

**Figure 15:**
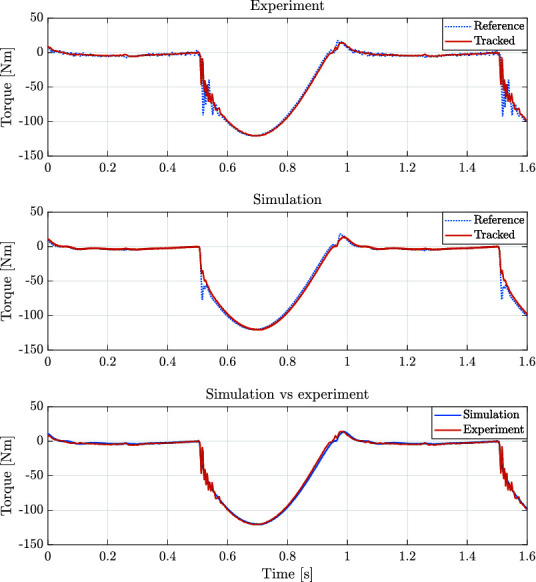
Torque applied for both simulation and experiment for the experiments corresponding to the impacts against the mechanical end-stop.

To evaluate the performance of the rotary actuator against impacts, we provided a periodic reference trajectory that results in continuous impacts against the mechanical end-stop. An example of these tests in simulation and experiments is shown in Figure 15. It can be seen that the magnitude of the applied torque in order to cope with this impulse-like disturbances remains within the actuator limits (i.e., 170 Nm). These results show that the hydraulic servo actuators are able to deal with this kind of perturbation remaining stable and avoiding saturation. It is also important to point out that the reference signal in the case of the experiment shows some spikes after the impact. These sudden increments in the reference are mainly originated from the velocity measurements and the rotor velocity error at the beginning of the contact.

**Figure 16:**
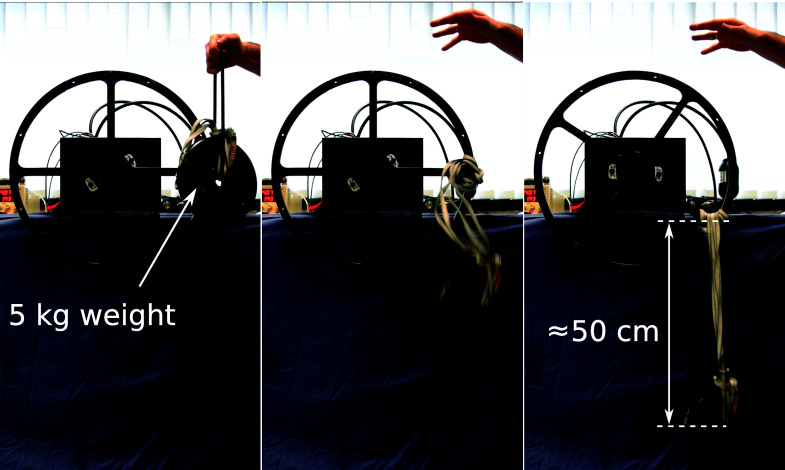
Experimental setup procedure taken to introduce external disturbances on the system. The figures are snapshots of three different moments that show (from the left to the right): the initial height of the 5 kg at the release phase; the weight in free fall; and the weight height in steady state. The rope length allows for a free fall of about 50 cm, leading to an impact velocity about 3.13 m/s and weight momentum about 15.66 kgm/s.

**Figure 17:**
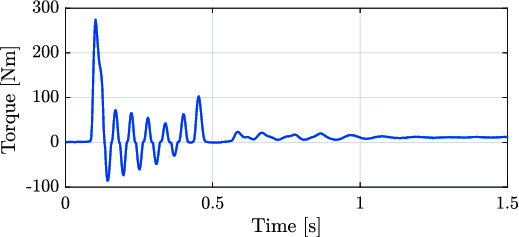
Recorded experimental data of the applied torque while dropping a 5 kg weight attached to the experimental setup of the rotary actuator with no torque control implemented.

**Figure 18:**
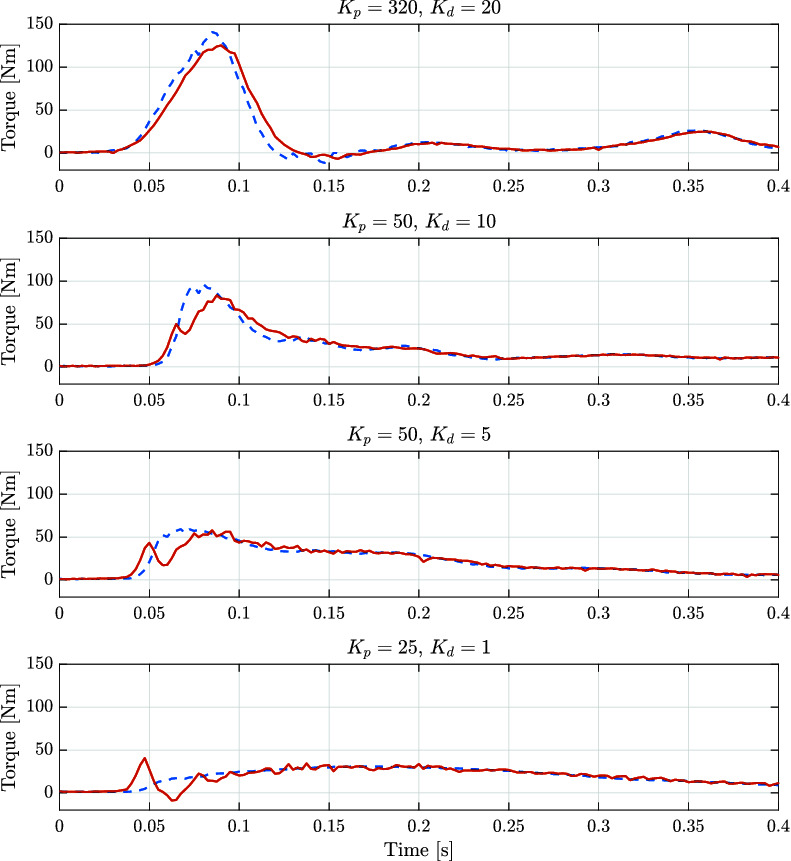
Recorded experimental data of the reference (dashed blue lines) and applied (solid red lines) torques while dropping a 5 kg weight attached to the experimental setup of the rotary actuator with different gains for the impedance control.

The final experiment for the rotary actuator is designed to emulate the presence of external disturbances into the system. The perturbation is introduced by attaching a weight to the edge of the load wheel of the experimental setup. A sequence of snapshots of this experiment is shown in Figure 16. The weight and rope slack used to apply the external disturbance could lead to a torque disturbance impulse with an amplitude larger than 250 Nm if the wheel was perfectly blocked or its joint had infinite joint stiffness. Figure 17 shows such large impacts considering the case where the system has load position control without impedance torque control. For the test shown in Figure 17 we consider a pure position control where the output of the position PID controller block $F_{r}$ becames the direct command signal $U_{v}$ to the hydraulic valve (see Figure 7A). As it can be seen, when torque control is not implemented, the time that the system takes to stabilize is much larger. On the other hand, when torque control is applied (as depicted in Figure 18), steady-state is reached much faster, with a lower level of oscillations.

Additionally, we tested our control scheme for different values of controller gains. This set of gains is considered to contain a range of impedances suitable for quadruped robot locomotion. Based on our experience with the HyQ-series robots, depending on the application, these values of impedances could be implemented in systems ranging from 80 kg to 120 kg. It can be seen that even with a compliant set of gains, the actuator is able to cope with a disturbance. These experiments show the relevance of implementing torque and impedance control when dealing with impacts.

Studying the comparison between the experimental data and the simulation, the proposed setup proved to be useful for controller design, since the same strategy with the same control parameters (controller gains and frequencies of the control loops) were chosen with similar results in performance. Added to the fact that the designed smart actuators presented in this paper fulfill the requirements of legged systems, it has been shown that the integration of the electronics and hydraulic actuation aids greatly in the design of new control strategies for hydraulic systems.

### 5.2. ISA V5 Driving Knee Joint of HyQ-REAL

For the linear actuator experiments, the ISA v5 was mounted on the KFE joint of the experimental setup for the leg of the hydraulically actuated robot HyQ-REAL. Position control and force tracking were also tested. Due to time constraints, a detailed simulation study of the implementation of the linear smart actuator was not carried out along with the experiments, mainly because additional experiments are required in order to properly identify the parameters of the leg. Nevertheless, the tests presented in this section contributed not only in the evaluation of the performance of the ISA v5, but also gave important insight about key parameters, such as the bandwidth of the system. It remains as future work to develop a simulation environment for behavior analysis and controller design, such as the one developed for the smart manifold integrated with the custom made rotary actuator.

**Figure 19:**
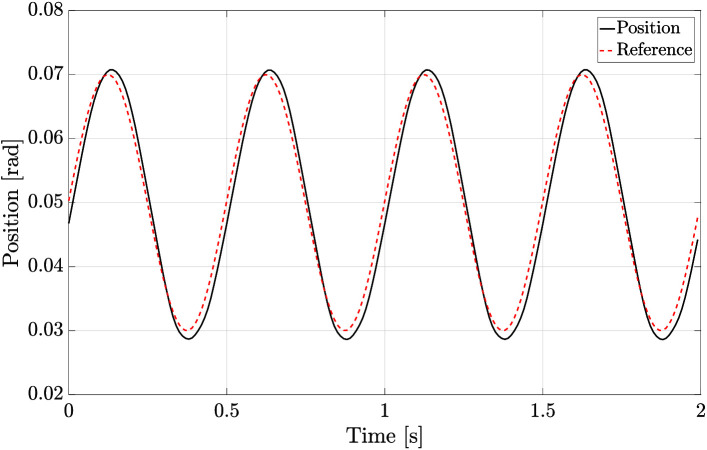
Position tracking of a sinusoidal wave by the KFE joint of the experimental setup for a leg of HyQ-REAL. The joint is actuated using the ISA v5.

Similarly to the position tracking experiment performed with the rotary actuator, a sine wave signal was set as reference for the enconder position of the KFE. Figure 19 shows the tracking performance of the system. Implementing the control strategy described in Figure 7, it can be seen that the tracking is similar to the one achieved with the rotary actuator.

The second experiment for the linear actuator is similar to the force control against the end-stop for the rotary actuator. In the case of the leg, the foot was put in contact with a wooden pallet (as it can be seen in the lower part of the picture displayed in Figure 10B) and a step reference for force was given, for low and high force values. The results of these experiments can be seen in Figure 14.

**Figure 20:**
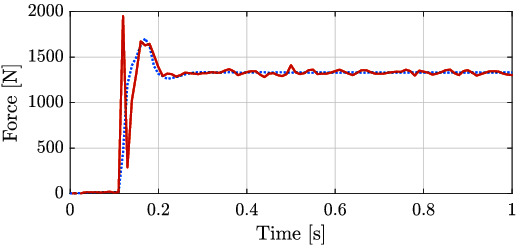
Force tracking of the ISA after the leg dropping from a 10 cm height. The approximate weights of the leg and the moving section of the slider are 10.2 kg and 5 kg, respectively. Reference is represented by the dotted line and applied torque corresponds to the red solid line.

For the last experiment, the leg was dropped from a height of 10 cm in order to evaluate the performance of the ISA when subjected to critical impacts, generally present while executing locomotion tasks. The total weight of the leg is approximately of 10.2 kg and the moving part from the slider that is attached to the leg (see Figure 10B) weighs approximately 5 kg. For this experiment the impedance values were $K_{p}=100000$ N/m and $K_{d}=4000$ Ns/m. Figure 20 shows the force tracking during this experiment. The impact of the leg takes place around 0.1, at which the reference signal sent by the position control loop starts being tracked by the force loop. It can be noticed, that the actuator is able to stabilize the leg, considerably below its maximum output force (7500 N). The size of the peak appearing right after the time of the impact is due to the time the system takes to perceive and respond against the external disturbance (the ground reaction force).

The results of the experiments show a similar behavior as the ones displayed by the rotary actuator for similar tests, after tuning the controllers in order to account for the dynamics of the leg. This represents a positive result towards the implementation of a systematic method to design, test and implement controllers for legged systems in the future. Regarding this last point, one of essential task for further research, is to take advantage of the possibility to provide a detailed model of the integrated actuators shown in this paper, and develop control strategies that could improve the performance based on these models such as the feedback linearization (Boaventura et al., 2015).

## 6. Discussion and Conclusions

This paper presented a detailed description of the Integrated Servo Actuators ISA v2, ISA v5 and the smart manifold integrated with a custom made rotary actuator, developed by Moog and IIT. The main features and specifications of the components that build up the system were conceived in order to satisfy the requirements of legged systems and to overcome the current shortcomings of hydraulically actuated robots. The devices here described successfully integrate the electronics for controller implementation along with the components that comprise a high-performance hydraulic actuation system.

A detailed dynamic model of the actuator is given and was verified to be representative after comparing the simulation and the experimental results. The main goals of providing such dynamic model and a detailed parametrization of the actuator features are: to help on the elaboration of new control strategies; to make the simulation of multi-legged systems more realistic by integrating the actuator dynamics; to help designing mechanical parts by foreseeing the impact of their geometric tolerances (e.g., that lead to backlash) and properties (e.g., that lead to friction or structure stiffness).

Differences in the results between experimental and simulated tests are expected due to the difficulty to model the fast and nonlinear dynamics present in the systems. Sources of mismatching dynamics are likely to be due to, e.g.: inaccuracies in the environment stiffness model; the asssunption of a test bench stiffness infinitly rigid; the nonlinearities of the valve around the spool null position that can not be precisely modeled; and the innacuracies in the friction modeling. Most of these modeling mismatching and assumptions tend to cause a difference between simulation and experimental results when the actuator performs velocities close to zero (condition where friction modeling is critical) or goes into a steady state where the valve spool tends to be positioned around null. At this critical conditions, high controller gains might lead to limit cycles as a steady state.

A control strategy implemented on the integrated electronic boards of the servo actuators is explained in detail. This control strategy was tested on two experimental setups and the experimental results for the smart manifold integrated with the rotary actuator and the ISA v5 mounted on the knee joint of HyQ-REAL leg were presented. The force/torque control performance, shown in Sec. 5, suggests a control bandwidth between 50 Hz and 100 Hz for low amplitude signals (about 10% of the maximum output force/torque). Considering the control bandwidth found in state-of-the-art papers for related applications (Paine et al., 2014; Boaventura et al., 2015; Hutter et al., 2016), that ranges from 30 Hz to 60 Hz for small signals around 10%, the control performance obtained with the smart actuarors is relevant, promissing and are part of the current state-of-the-art.

In its current state, the main limitations noticed in the smart actuators are at firmware level. Future work includes implementing new functionalities to test different control strategies (e.g., nonlinear control approaches) as well as tools to run system identification algorithms and to evaluate the control performance of the various control loops (e.g., frequency response analysis).

Future work also comprises new steps towards the system modeling. A detailed characterization for simulation and controller design of the experimental setup of the ISA v5 along with the HyQ-REAL leg will be obtained. Moreover, different control strategies that might improve the performance of the overall system (such as model based strategies like feedback linearization) will be tested both in simulation and experiments. Finally, the novel servo actuators here described, will be implemented on HyQ-REAL, the newest version of the hydraulically actuated quadrupeds developed in IIT.

## Author Contributions

VB, DS, MF, MB, RM contributed to the development of the actuators and setups. VB, OV-M, DS designed and performed the experiments. VB, OV-M and GM-C did the modeling part and performed the simulations. CS, MB and DC coordinated the teams. VB, OV-M, DS and CS prepared the manuscript and figures.

## Conflict of Interest Statement

Authors DS, MB and RM are employed by company Moog (UK). All other authors declare no competing interests and have no involvement or role in the company.

## Footnotes

1. http://www.moog.com/content/dam/moog/literature/ICD/Moog-Valves-30series-datasheet-en.pdf

2. The most recently presented *SpotMini* is BDI’s smallest and lightest (30 kg) quadruped and it uses electric actuation.

3. http://www.moog.com/literature/ICD/e024seriesmicrovalves-ds.pdf

4. http://www.psc-net.co.jp/category/1359255.html

5. http://www.moog.com/products/actuators-servoactuators/industrial/hydraulic/ a085-series-hydraulic-servo-actuators.html

6. http://rnd.knrsys.com/english/view.html?id_no=48

7. http://www.star-hydraulics.co.uk/assets/data-docs/product%20pdfs/ST-200-2017-2-En.pdf

8. An extended version of this section will be published in the Springer book *Humanoid Robotics: A Reference* in the chapter *Actuator Modeling and Simulation* by Jorn Malzahn, Victor Barasuol, and Klaus Janschek.
